# Impact of COVID-19 Pandemic on Healthcare Utilization in People with Diabetes: A Time-Segmented Longitudinal Study of Alberta’s Tomorrow Project

**DOI:** 10.3390/healthcare12192009

**Published:** 2024-10-08

**Authors:** Ming Ye, Jennifer E. Vena, Grace Shen-Tu, Jeffrey A. Johnson, Dean T. Eurich

**Affiliations:** 1 School of Public Health, University of Alberta, Edmonton, AB T6G 2G4, Canada; 2 Alberta’s Tomorrow Project, Cancer Care Alberta, Alberta Health Services, Calgary, AB T2T 5C7, Canada

**Keywords:** diabetes, COVID-19, healthcare utilization, public health emergency

## Abstract

Objective: The objective is to characterize the impact of COVID-19 on major healthcare for diabetes, including hospitalization, emergency department (ED) visits and primary care visits in Alberta, Canada. Methods: Participants from Alberta’s Tomorrow Project (ATP) with pre-existing diabetes prior to 1 April 2018 were included and followed up to 31 March 2021. A time-segmented regression model was used to characterize the impact of COVID-19 on healthcare utilization after adjusting for seasonality, socio-demographic factors, lifestyle behaviors and comorbidity profile of patients. Results: Among 6099 participants (53.5% females, age at diagnosis 56.1 ± 9.9 y), the overall rate of hospitalization, ED visits and primary care visits was 151.5, 525.9 and 8826.9 per 1000 person-year during the COVID-19 pandemic (up to 31 March 2021), which means they reduced by 12% and 22% and increased by 6%, compared to pre-pandemic rates, respectively. Specifically, the first COVID-19 state of emergency (first wave of the outbreak) was associated with reduced rates of hospitalization, ED visits and primary care visits, by 79.4% (95% CI: 61.3–89.0%), 93.2% (95% CI: 74.6–98.2%) and 65.7% (95% CI: 47.3–77.7%), respectively. During the second state of emergency, healthcare utilization continued to decrease; however, a rebound (increase) of ED visits was observed during the period when the public health state of emergency was relaxed. Conclusion: The declared COVID-19 states of emergency had a negative impact on healthcare utilization for people with diabetes, especially for hospital and ED services, which suggests the importance of enhancing the capacity of these two healthcare sectors during future COVID-19-like public health emergencies.

## 1. Introduction

At the early stage of the COVID-19 outbreak (March 2020), provincial and territorial governments in Canada, including the Government of Alberta, declared states of public health emergency and implemented public health measures, including physical distancing, wearing masks in public places and closure/suspension of non-essential business/services to mitigate the spread of COVID-19 [1]. In Alberta, to ensure capacity of the healthcare systems to handle the COVID-19 emergency, Alberta Health Services (AHS), which delivers healthcare services throughout the province, reconfigured Alberta’s healthcare delivery, including developing protocols for COVID-19 testing, maintaining robust inventories of supplies for the COVID-19 emergency and reducing and suspending non-urgent care and non-essential health services [2].

Nevertheless, the adaptation of existing healthcare systems to the COVID-19-focused agenda may have disrupted regular healthcare in Canada. It has been reported that in 2020, emergency department (ED) visits dropped by 25–50% in Canada [3]. A population-based study showed that hospital admission and ED visits decreased by approximately 40% in patients with heart failure in Toronto in 2020 compared to the year before [4]. Reports from AHS also highlight a remarkable increase in backlogged health services, including regular checkups, clinical tests, as well as outpatients services for “non-urgent” conditions during COVID-19 outbreaks in Alberta [2]. Several studies in the U.S. and Canada have also indicated that the COVID-19-related delays in health services may have contributed to increased morbidity and mortality for patients with pre-existing conditions, including diabetes [5].

In fact, with COVID-19 infection, patients with diabetes often experience a higher mortality rate than patients without diabetes [6]. A recent global survey from healthcare professionals also indicates that patients with diabetes were the patients most influenced by a reduction in healthcare resources during the COVID-19 pandemic [7]. However, little is known on the exact impact of COVID-19 on healthcare utilization in people with diabetes in Canada, especially given healthcare systems and patients experiencing episodic changes in public health regulations during this period. In this study, we used Alberta’s Tomorrow Project (ATP) data, a population-based cohort study focusing on chronic diseases in Alberta, Canada, linked with individual-level healthcare databases, to characterize the impact of COVID-19 states of emergency on major health services utilization, including all-cause hospitalization, ED visits and primary care visits, in people with diabetes. Our study not only characterized the impact of COVID-19 on healthcare in patients with diabetes but also reflected the influence from the COVID-19 pandemic on the health and wellbeing of the Canadians. Moreover, unlike previous studies, we have incorporated into our analysis many difficult-to-capture patient factors, including socio-economic factors, anthropometrics (e.g., BMI) and lifestyle behaviors (e.g., dietary intake and physical activities) which are well known to influence healthcare utilization in patients with diabetes.

## 2. Materials and Methods

### 2.1. Study Design

We used data from ATP, linked with administrative healthcare data, to conduct an individual-level, time-segmented longitudinal study to characterize the impact of COVID-19 states of emergency on all-cause hospitalization, ED visits and primary care visits in patients with diabetes.

### 2.2. Study Participants

As described previously, from 2000 to 2015, ATP recruited ~55,000 participants from the adult population (35–69 years of age) in Alberta with cancer other than non-melanoma skin cancer at enrollment [8]. All ATP participants answered the ATP Health and Lifestyle Questionnaire (HLQ), and 99% consented to data linkage to Alberta Health (AH) administrative healthcare data. This study includes ATP participants with diabetes diagnosed prior to 1 April 2018, i.e., approximately two years before the initial outbreak of COVID-19 in Alberta, which allows sufficient data points for estimating potential seasonal patterns of healthcare utilization.

### 2.3. Data Sources

This study utilized Alberta Health (AH) administrative healthcare data, including ambulatory care data (i.e., ED visits), inpatient data, physician claims data and drug dispenses data. Descriptions of AH healthcare data can be obtained from https://open.alberta.ca/dataset/overview-of-administrative-health-datasets (accessed on 4 October 2024. Individual-level AH data up to 31 March 2021 (the latest AH data available when the study was conducted) were linked to the ATP cohort using participants’ Personal Health Numbers (PHNs) provided during the consent process. Additional AH data were also linked retrospectively to 1 April 2000 for identifying all existing cases of diabetes.

In addition to healthcare data, a wide range of information related to chronic diseases, including sociodemographic and socio-economic factors, anthropometrics and lifestyle behaviors (e.g., dietary intake and physical activities), were provided by the ATP participants at the time of enrollment using the ATP health and lifestyle questionnaire, Canadian Dietary History Questionnaire (CDHQ) and Past-Year Total Physical Activity Questionnaire (PYTPAQ). Detailed information can be found at www.myATP.ca (accessed on 4 October 2024).

### 2.4. Diabetes Cases

The ATP participants with diabetes prior to 1 April 2018 were identified from the total ATP cohort using the linked healthcare data and a modified algorithm from the Canadian National Diabetes Surveillance System (NDSS):

“one hospitalization record with an ICD code of diabetes (ICD-9: 250, ICD-10: E10–E14) OR two physician claims within two years with an ICD code of diabetes OR self-report by participants, plus any of the following conditions: (i) one hospitalization with ICD code for diabetes, (ii) one physician claim with ICD code for diabetes, or (iii) one diabetes medication with Anatomical Therapeutic Chemical Classification (ATC) code for insulin (A10A) or glucose-lowering drugs (A10B)”

[9]

The index date of diabetes was the earliest date of clinical records that contribute to the case definition.

### 2.5. COVID-19 States of Emergency

The study period (1 April 2018–31 March 2021) includes two declared COVID-19 states of emergencies (17 March 2020–15 June 2020 and 27 November 2020–22 February 2021) [10] and a “relaxation” period between the two emergency states. In the analyses, 157 time segments (weeks 1–157) were defined for the whole study period based on calendar week, and the two COVID-19 states of emergency were in weeks 103–116 (17 March 2020–15 June 2020) and weeks 140–152 (27 November 2020–22 February 2021), respectively.

### 2.6. Outcomes of Interest

The outcome of interest was rate (density) of health services utilization, including primary care visits, hospitalization and ED visits. Primary care visits were identified based on records of visits to clinicians [general practitioner (GP) or specialist] in an outpatient environment (e.g., physician’s office) in physician claims data; hospitalization were identified based on the DAD data; ED visits were identified based on ambulatory care records with Alberta Management Information System (MIS) codes of 71310, 71313 and 71314); virtual visits to a GP or specialist were identified by the billing codes (03.01AD, 03.03CV, 03.03FV, 03.08CV, 03.05JR, 03.01S, 03.01T) in physical claims data for virtual care (i.e., via telephone, email and/or videoconference).

### 2.7. Statistical Analysis

We used the standard exploratory data analysis method to summarize study participants and rates of healthcare utilization, i.e., means and standard deviations were calculated for continuous variables; proportions were calculated for categorical variables; rates (density) were calculated for person-year data. Comparisons between groups were made using Student *t*-test for continuous variables and *x*^2^ test for categorical variables and cumulative rates.

The rate (density) of healthcare utilization was defined as a ratio of the number of healthcare visits divided by the total person-years (PY) for a certain period, until the end of study (31 March 2021), a migration out of the province (based on Alberta Population Registry data) or a death (based on Alberta Vital Statistics data). The rates of healthcare utilization were calculated (i) for the period before (1 April 2018–16 March 2020) vs. during (17 March 2020–31 March 2021) the COVID-19 pandemic; (ii) for each of the two COVID-19 states of emergency and the “relaxation” period (i.e., the period between the two states of emergency); and (iii) for each calendar week (weekly rate) as an outcome variable in time-segmented regression analyses.

The impact of COVID-19 states of emergency on healthcare utilization was characterized by a time-segmented (piece-wise) generalized linear regression model (GLM) with individual-level data [11]. With the GLM model, the percentage of changes in healthcare utilization (transformed from changes in log rate, and the +/− signs of changes in log rate suggest increase/decrease in healthcare utilization), and their 95% CIs were computed after adjusting for the within-subject correlation of weekly repeated measures of healthcare utilization over time and the following covariates: seasonality, age at diagnosis (35–44/45–64/65+ years), sex (male, female), ethnicity (European ancestry vs. other), rural vs. urban areas, education (secondary or less/some post-secondary/post-secondary), body mass index (BMI) categories (≤24.9 kg/m^2^, 25.0–29.9 kg/m^2^, ≥30.0 kg/m^2^), ever smoker (yes/no), physically active (yes/no, based on accumulating at least 210 min of moderate- to vigorous-intensity recreational physical activities per week in the past 12 months) [12], tertiles of the 2005 Canadian Healthy Eating Index (HEI) for diet quality assessment [13] and the number of Elixhauser comorbidities (0, 1–2, 2+) at diagnosis [14]. For participants with missing values in these covariates, a “missing” category was created for these patients to ensure inclusion of all participants, and thus results were minimally affected. Full details of the model can be found in Supplementary Table S1. Statistical analyses were conducted using STATA^®^14, at significant level of 0.05.

## 3. Results

### 3.1. Characteristics of Study Participants

Overall, 6099 ATP participants with diabetes diagnosed prior to 1 April 2018 were identified and included in the study. The average age at diagnosis was 56.1 ± 9.9 years, and 53.5% of the participants were women. The majority (80.0%) of participants self-reported as being of European ancestry, and 21.4% of participants lived in rural areas. At the time of diagnosis, approximately 20% of participants had 2+ Elixhauser comorbid conditions (Table 1).

### 3.2. Healthcare Utilization before and within COVID-19 Pandemic

During the entire study period of 157 weeks, the average (cumulative) rates of hospitalization, ED visits and primary care visits for patients with diabetes were 165.4, 619.9 and 8502.6 per 1000 person-year, respectively. During the COVID-19 pandemic (17 March 2020 to 31 March 2021), the average rates of hospitalization and ED visits were 151.5 and 525.9 per 1000 person-year, respectively, which is a decrease of 12% (95% CI: 5–19%) and 22% (95% CI: 18–25%), respectively, compared to the pre-pandemic rates (Table 2). The average rate of primary care visits was 8826.9 per person-year during the COVID-19 pandemic, an increase of 6% (95% CI: 5–7%) compared to the pre-pandemic rate. In addition, the average rate of virtual care visits was 3975.2 per person-year during the COVID-19 pandemic, 15.61 times (95% CI: 15.03–16.21) greater than the rate before the pandemic (254.7 per person-year) (Table 2).

When breaking down the COVID-19 pandemic into the two states of emergency and a “relaxation” period, the rate of hospitalization decreased in both states of emergency, with rate ratios of 0.78 (95% CI: 0.67–0.90) and 0.81 (95% CI: 0.69–0.94), respectively, compared to the pre-pandemic. During the “relaxation” period, the rate of hospitalization was similar to the rate before the COVID-19 pandemic [rate ratio = 1.02 (95% CI: 0.92–1.13)] (Table 2). However, compared to the period before the COVID-19 pandemic, the rates of ED visits decreased in all periods (irrespective of whether the COVID-19 state of emergency was relaxed), with rate ratios of 0.70 (95% CI: 0.65–0.76), 0.87 (95% CI: 0.83–0.92) and 0.73 (95% CI: 0.67–0.79), respectively. Compared to the pre-pandemic rate, the rate of primary care visits remained unchanged (rate ratio = 0.99, 95% CI: 0.98–1.02) during the first COVID-19 states of emergency, but increased during the “relaxation” period and the second state of emergency, with rate ratio of 1.10 (95% CI: 1.08–1.11) and 1.05 (95% CI: 1.03–1.07), respectively (Table 2).

### 3.3. Impact of COVID-19 on Health Services Utilization

**Figure 1:**
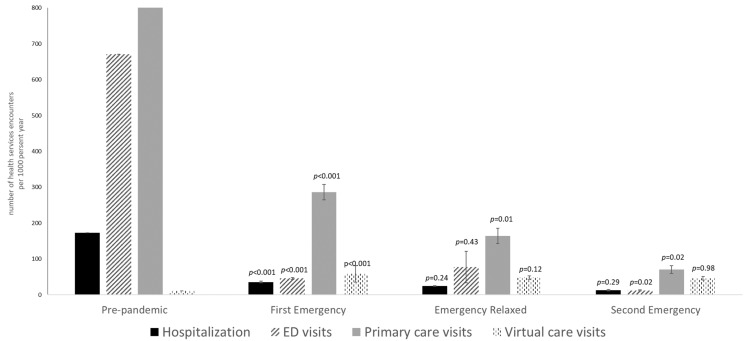
Model-based rates of health services utilization before and during COVID-19 states of emergency: results from multivariable regression analysis.

In the multivariable regression model, during the first COVID-19 state of emergency, the rate of hospitalization decreased by 79.4% (95% CI: 61.3–89.0%), ED visits by 93.2% (95% CI: 74.6–98.2%) and primary care visits by 65.7% (95% CI: 47.3–77.7%), and the virtual care visits increased by 552.9% (95% CI: 263.8–1102.3%), compared to the pre-pandemic rates (Figure 1). In the “relaxation” period, the rate of hospitalization and primary care visits continued to decrease by 31.6% (95% CI: −63.9%, 28.4%) and 42.9% (95% CI: −63.2%, −10.4%), respectively; however, the rate of ED visits increased by 69.9% (95% CI: −54.2%, 529.7%) compared to the first COVID-19 state of emergency. When the second COVID-19 state of emergency was in effect, health services utilization continuously decreased by 47.3% (95% CI: −84.0%, 73.3%), 84.3% (95% CI: −96.5%, −30.2%) and 56.8% (95% CI: −79.0%, −12.2%) for hospitalization, ED visits and primary care visits, respectively, compared to the previous “relaxation” period (Figure 1).

The estimated rates of healthcare utilization (per 1000 person-year for hospitalization and emergency department (ED) visits and per 10,000 person-year for primary care and virtual care visits) were calculated from the anti-log transformation of beta-coefficients in multivariable generalized linear regression models (GLM), after adjusting for seasonality, age, sex, ethnicity, living in rural vs. urban areas, education attainment, BMI categories, ever smoking (yes/no), physically active (yes/no), tertiles of the 2005 Canadian Healthy Eating Index, the number of Elixhauser comorbidity at diagnosis. The error bars represent the standard deviation of the estimated rates of healthcare utilization. Time periods were defined as pre-pandemic (1 April 2018–16 March 2020), first COVID-19 states of emergency (17 March 2020–15 June 2020), second COVID-19 states of emergency (27 November 2020–22 February 2021) and the period when states of emergency were relaxed (16 June 2020–26 November 2020), respectively. Comparisons (*p*-values) was made against the adjacent period.

In the subgroup analyses (Supplementary Table S2), the level of increase in virtual care visits during the first COVID-19 state of emergency was higher for diabetes patients living in urban areas compared to rural areas [log rate change = 1.31 (95% CI: 0.78–1.84) in urban vs. 0.88 (95% CI: −0.44–2.21) in rural, *p* = 0.03 for comparison]. In addition, during the first COVID-19 emergency, diabetes patients with complications had a higher level of reduction in hospital admission compared to those without complications [log rate change = −1.48 (95% CI: −2.15–0.82) with complications vs. −0.10 (95% CI: −2.24–2.04) without complications, *p* = 0.04 for comparison]. No statistically significant differences were observed between subgroups in other healthcare sectors during the COVID-19 pandemic (Supplementary Table S2).

## 4. Discussion

This individual-level, time-segmented longitudinal study shows that the COVID-19 states of emergency in Alberta were associated with significant reduction in health services utilization, including hospitalization, ED visits and primary care visits, in patients with diabetes. Although there was an increase in ED visits during the “relaxation” period, healthcare utilization in patients with diabetes did not fully rebound to pre-pandemic levels.

The significant reduction shown in our regression modeling of ED visits (80–90% reduction), hospital admission (50–80% reduction) and primary care visits (55–65% reduction) in patients with diabetes during the COVID-19 pandemic in Alberta is consistent with the world-wide fact that healthcare systems were quickly overloaded, especially for ED and hospital services during the first year of the COVID-19 pandemic [15]. A recent population-based study in Ontario, Canada, found similar results of disrupted hospital care and reduced ED visits in patients with diabetes [16]. Our study also found that after a significant reduction in ED visits during the first wave of the pandemic, there was a rebound (increase) of ED visits when the first COVID-19 state of emergency was relaxed in Alberta. Similar results were observed in other studies in Finland [17] and China [18]. This rebounding effect was likely due to a strong recovery of backlogged services during the “relaxation” period to accommodate episodic changes in public health regulations, including multiple cycles of public health “restriction/lockdown” and “relaxation/reopening” during the COVID-19 pandemic in Alberta [10]. For primary care, similar to other provinces in Canada [19,20], our study found a continuous reduction in primary care visits during the COVID-19 pandemic in Alberta, and certain public health measures (e.g., physical distancing) during the pandemic may have inadvertently reduced the willingness of some patients to visit family doctors routinely [1,21]. The lower rate of primary care visits during the COVID-19 pandemic might have led to a significant reduction in diabetes diagnoses in Canada [22]. Moreover, fewer primary care visits during the pandemic could be concomitant with a reduced number of prescriptions of anti-hyperglycemic drugs for patients with diabetes in Canada [23].

Patients with diabetes often require continuing care and timely treatment to achieve better outcomes [24,25]. The ATP COVID-19 Survey (unpublished data from https://myatp.ca/for-participants/covid-19-survey, accessed on 4 October 2024) suggests there were a large proportion of patients who did not seek healthcare for reasons including the perspective that health services were preserved for patients with severe COVID-19 infection and a fear of getting infected in public spaces. A few studies also showed that many patients with chronic diseases, including those with hypertension, cardiovascular diseases and diabetes, had to reschedule or cancel their regular checkups and follow-up visits during the COVID-19 pandemic [26,27]. In fact, compared to general populations, patients with pre-existing chronic diseases were at a higher risk of developing worse outcomes following a COVID-19 infection [28,29]. Our subgroup analysis showed that the reduction in hospital admission during the first COVID-19 emergency in Alberta was mainly from diabetes patients with complications, indicating Alberta was in need of adequate resources for hospital care to address future COVID-19-like public health emergencies for those high-risk populations. Our subgroup analysis also showed that, although not statistically significant, the level of reduction in all main healthcare sectors (primary care, ED, hospital) was higher for patients living in rural areas compared to urban areas, especially during the first wave of COVID-19 outbreak (Supplementary Table S2). Similar results were observed in the rates of hospitalization in the United States (U.S.) during the COVID-19 pandemic [30]. In addition, while our study showed that the pandemic-urged implementation of virtual care in Alberta had partially compensated for the pandemic impact on healthcare, the level of increase in virtual care visits in rural areas in Alberta significantly lagged behind urban areas at the beginning of the COVID-19 pandemic. This result is consistent with observations in a population-based study in the U.S. study showing that rural patients had fewer virtual care visits than those in urban areas, despite the increase in use of virtual care during the COVID-19 pandemic [31]. These results, along with our study, suggest re-configuration of healthcare delivery and the associated reduction in health services utilization during the COVID-19 pandemic might have exerted significant negative impacts on diabetes care and outcomes for these patients in Alberta. Future consideration of an effective but more balanced (for high-risk patients, including those with pre-existing chronic conditions and patients living in rural areas) system-level re-organizing or re-enforcing of health resources, especially for hospital and ED services, during COVID-19-like pandemics is necessary.

### Limitations

Our study had limitations. First, while our study participants were recruited from the general population in Alberta, the ATP participants may not be a representative sample of the general population in Alberta, Canada. When compared to the Canadian Community Health Survey (CCHS) participants, the ATP cohort had more women, with older ages and relatively healthier lifestyles, compared to the general population in Alberta [8]. These differences in study participants may have a mixed influence on our results, given a lower rate of diabetes in women and people with healthier lifestyles [32] but higher risk and more healthcare needs for patients with older ages [33]. Nevertheless, given the high internal validity (e.g., large number of participants and controlling for a wide range of individual-level confounders), our study provides a new piece of evidence on the negative impact of the COVID-19 pandemic on health service utilization in patients with diabetes, which to our knowledge is the first in Canada. Second, the provincial administrative healthcare data are not designed for the purpose of research, and measurement errors from un-reported, mis-reported or delayed reports [34] and/or coding mistakes (e.g., convenient coding and umbrella/incomplete coding) [35] may exist. While the administrative data are a reliable data source for health services research [36], in our secondary study of administrative health data, coding errors and/or incomplete records, especially from the early stage transition from regular care to a COVID-19-focused agenda in Alberta [2], may have influenced the level of health services during the pandemic. In addition, although we used a “missing indicator” category for self-reported covariates to mitigate the impact of missing data, study results may still be subject to information bias. Lastly, our analysis showed the impact of the first two COVID-19 emergencies (up to March 2021), which included the latest data available when our study was conducted. However, the data availability limits our ability to further assess the impact of the third (i.e., the last) COVID-19 state of emergency in Alberta (No state of emergency was declared nor were health interventions implemented for the fourth and further outbreaks) [10]. Future study using the updated healthcare data, including the third state of emergency and some post-pandemic data is warranted.

## 5. Conclusions

By linking individual-level health and lifestyle data of the ATP cohort to provincial administrative healthcare data, our study shows that the COVID-19 states of emergency in Alberta were associated with significant reduction in healthcare utilization in patients with diabetes. This negative impact was particularly evident for hospital and ED services. Future system-level public health interventions need to focus on improving the capacity of hospital and ED services to respond to largescale, COVID-19-like public health emergencies. Results of our study also suggest that more balanced public health response strategies are necessary during future health emergencies, especially at the early stage (e.g., the first wave of the COVID-19 outbreak) for patients with high-risk health conditions (e.g., diabetes with complications).

## Figures and Tables

**Table 1:** Characteristics of study participants.

		Female (n = 3262, 53.5%)	Male (n = 2837, 46.5%)	Total (n = 6099, 100%)
		Mean (SD), %	Mean (SD), %	Mean (SD), %
Age at diagnosis *				
years		55.4 (10.4)	57.0 (9.1)	56.1 (9.9)
Ethnicity				
European ancestry		79.2	80.9	80.0
Other		11.6	11.4	11.5
Missing		9.2	7.7	8.5
Rural/urban *				
Rural		21.9	20.7	21.4
Urban		78.1	79.3	78.6
Education level *				
High school or less		33.7	29.5	31.8
Some post-secondary		47.3	45.6	46.5
Post-secondary		19.0	24.9	21.7
BMI category *				
≤24.9 kg/m^2^		7.9	5.5	6.8
25.0–29.9 kg/m^2^		19.9	27.2	23.3
≥30.0 kg/m^2^		55.2	53.5	54.4
Missing		17.1	13.7	15.5
Ever smoking *				
No		43.3	33.4	38.6
Yes		56.7	66.6	61.4
Physically active ^a^				
No		42.5	41.5	42.0
Yes		37.5	33.0	35.4
Missing		20.1	25.5	22.6
Diet quality ^b^				
Average score *		55.1 (9.9)	50.7 (9.0)	53.0 (9.7)
Lowest tertile		16.0	26.5	20.9
Medium tertile		18.9	19.7	19.2
Highest tertile		23.0	12.6	18.2
Missing		42.2	41.2	41.7
Number of comorbidities at diagnosis				
Average *		1.6 (1.6)	1.4 (1.7)	1.5 (1.6)
0		22.7	29.2	25.8
1–2		57.0	55.2	56.1
2+		20.3	15.6	19.1

^a.^ Categories (Yes/No) were created according to accumulating at least 210 min of moderate- to vigorous- intensity recreational physical activities per week in the past 12 months; ^b.^ Scores were determined from the Canadian Diet History Questionnaire and using the 2005 Canadian Healthy Eating Index. * Statistically significantly different (*p* < 0.05) across groups (female vs. male). Abbreviations: BMI = body mass index; SD = standard deviation.

**Table 2:** Unadjusted rates of health services utilization before and during the COVID-19 pandemic.

(n = 6099)	1 April 2018–16 March 2020 (before the Pandemic)	17 March 2020–31 March 2021 (during the Pandemic)			Entire Period
Person-Year	11,922.5	6428.8			18,351.3
hosp.	2062	974			3036
Rate *	173.0	151.5			165.4
Rate ratio **	Ref.	0.88 (0.81–0.95)			-
ED visits	7995	3381			11,376
Rate *	670.6	525.9			619.9
Rate ratio **	Ref.	0.78 (0.75–0.82)			-
Primary care visits	99,287	56,746			156,033
Rate *	8327.7	8826.9			8502.6
Rate ratio **	Ref.	1.06 (1.05–1.07)			-
Virtual care visits	3037	25,556			28,593
Rate *	254.7	3975.2			1558.1
Rate ratio **	Ref.	15.61 (15.03–16.21)			-
		**17 March 2020–15 June 2020** **(Emerg. 1)**	**16 June 2020–26 November 2020** **(Emerg. Relaxed)**	**27 November 2020–22 February 2021** **(Emerg. 2)**	
Person-Year	11,922.5	1519.5	2805.3	1402.6	18,351.3
hosp.	2062	204	496	196	3036
Rate *	173.0	134.3	176.8	139.7	165.4
Rate ratio **	Ref.	0.78 (0.67–0.90)	1.02 (0.92–1.13)	0.81 (0.69–0.94)	-
ED visits	7995	715	1638	687	11,376
Rate *	670.6	470.5	583.8	489.8	619.9
Rate ratio **	Ref.	0.70 (0.65–0.76)	0.87 (0.83–0.92)	0.73 (0.67–0.79)	-
Primary care visits	99,287	12,649	25,581	12,288	156,033
Rate *	8327.7	8324.3	9118.8	8760.6	8502.6
Rate ratio **	Ref.	0.99 (0.98–1.02)	1.10 (1.08–1.11)	1.05 (1.03–1.07)	-
Virtual care visits	3037	7391	9893	5823	28593
Rate *	254.7	4864.0	3526.6	4151.4	1558.1
Rate ratio **	Ref.	19.09 (18.30–19.93)	13.84 (13.29–14.42)	16.30 (15.60–17.03)	-

* Per 1000 PY. ** The rate before COVID-19 (1 April 2018–16 March 2020) as a reference.

## Data Availability

Data are contained within the article or Supplementary Materials. To comply with the Alberta Health (AH) Health Information Policy and Alberta’s Tomorrow Project data disclosure guidelines, datasets used in this study are not available for readers. Access to individual-level data is available in accordance with the Health Information Act of Alberta and the Alberta’s Tomorrow Project (ATP) Access Guidelines and Procedures. More information can be obtained via https://myatpresearch.ca (accessed on 4 October 2024).

## Supplementary Materials

The following supporting information can be downloaded at https://www.mdpi.com/article/10.3390/healthcare12192009/s1: Table S1: The COVID-19 states of emergency and rates of health services utilization; Table S2: The impact of COVID-19 emergency on health services utilization: results from stratified analyses.
