# Supplementary material for: Impact of COVID-19 Pandemic on Healthcare Utilization in People with Diabetes: A Time-Segmented Longitudinal Study of Alberta’s Tomorrow Project
Source: Healthcare (Basel). 2024 Oct 8;12(19):2009. doi: 10.3390/healthcare12192009 (PMC11476217; doi:10.3390/healthcare12192009)
Supplement: Supplementary file 1 [file healthcare-12-02009-s001.zip › healthcare-3194472-supplementary.pdf]

Supplementary Materials

Table S1. The COVID-19 states of emergency and rates of health services utilization: results from multivariable regression model (n = 6099)

	β coef.*	95% CI	P-value
**Hospitalization**			
pre-pandemic trend in rate	-0.038	-0.039, -0.037	<0.001
**Emerg. 1: March 17, 2020-June 15, 2020**			
change in log rate	-1.58	-2.21, -0.95	<0.001
actual trend in log rate	-0.011	-0.017, 0.005	<0.001
change in trend **	0.027	0.021, 0.032	<0.001
**Emerg. relaxed: June 16, 2020-November 26, 2020**			
change in log rate	-0.38	-1.02, 0.25	0.24
actual trend in log rate	-0.010	-0.014, -0.007	<0.001
change in trend **	0.001	-0.005, 0.007	0.83
**Emerg. 2: November 27, 2020-February 22, 2021**			
change in log rate	-0.64	-1.83, 0.55	0.29
actual trend in log rate	-0.010	-0.018, -0.002	0.016
change in trend**	0.0004	-0.008, 0.009	0.93
**ED visits**			
pre-pandemic trend in rate	-0.030	-0.031, -0.029	<0.001
**Emerg. 1: March 17, 2020-June 15, 2020**			
change in log rate	-2.69	-4.01, -1.37	<0.001
actual trend in log rate	0.001	-0.011, 0.014	0.85
change in trend **	0.031	0.019, 0.044	<0.001
**Emerg. relaxed: June 16, 2020-November 26, 2020**			
change in log rate	0.53	-0.78, 1.84	0.43
actual trend in log rate	-0.007	-0.012, -0.003	0.003
change in trend **	-0.008	-0.022, 0.005	0.21
**Emerg. 2: November 27, 2020-February 22, 2021**			
change in log rate	-1.85	-3.34, -0.36	0.015
actual trend in log rate	0.002	-0.008, 0.012	0.75
change in trend**	0.009	-0.002, 0.02	0.10
**Primary care visits**			
pre-pandemic trend in rate	-0.021	-0.022, -0.021	<0.001
**Emerg. 1: March 17, 2020-June 15, 2020**			
change in log rate	-1.07	-1.50, -0.64	<0.001
actual trend in log rate	-0.009	-0.013, -0.005	<0.001
change in trend **	0.012	0.008, 0.016	<0.001
**Emerg. relaxed: June 16, 2020-November 26, 2020**			
change in log rate	-0.56	-1.00, -0.11	0.014
actual trend in log rate	-0.006	-0.008, -0.004	<0.001
change in trend **	0.003	-0.001, 0.007	0.16
**Emerg. 2: November 27, 2020-February 22, 2021**			
change in log rate	-0.84	-1.56, -0.13	0.02
actual trend in log rate	-0.003	-0.007, 0.002	0.24

change in trend**	0.003	-0.002, 0.008	0.23
**Virtual care visits**			
pre-pandemic trend in rate	0.023	0.021, 0.026	<0.001
**Emerg. 1: March 17, 2020-June 15, 2020**			
change in log rate	1.71	0.97, 2.40	<0.001
actual trend in log rate	0.023	-0.001, 0.047	0.06
change in trend **	0.00002	-0.024, 0.024	0.98
**Emerg. relaxed: June 16, 2020-November 26, 2020**			
change in log rate	-0.19	-0.44, 0.05	0.12
actual trend in log rate	0.004	-0.005, 0.012	0.39
change in trend **	-0.019	-0.044, 0.005	0.12
**Emerg. 2: November 27, 2020-February 22, 2021**			
change in log rate	-0.044	-0.60, 0.51	0.98
actual trend in log rate	0.008	-0.023, 0.039	0.62
change in trend**	0.004	-0.027, 0.036	0.79

* beta coefficient (change in log rate) was estimated using multivariable generalized linear regression models (GLM), after adjusting for seasonality (cos, sin), age, sex, ethnicity, living in rural vs. urban areas, education attainment, BMI categories, ever smoking (yes/no), physically active (yes/no), tertiles of the 2005 Canadian Healthy Eating Index, and the number of Elixhauser comorbidity at diagnosis.

** rate/trend in the current time segment as opposed to the adjacent previous time segment

Detailed description of regression model:

We used the following time-segmented (piece-wise) regression model with individual-level data to characterize the impact of COVID-19 emergencies (defined by time segments) on healthcare utilization:

$$\log(Y_{wk}) = \beta_0 + \beta_1 * wk + \beta_{2i} * seg\__i + \beta_{3i} * wk\_in\_seg\__i + \beta_k * X_k + error \qquad \textbf{(Model 1)}$$

We assumed that a linear relationship between time (calendar weeks), the COVID-19 states of emergency as time segments of interest and the rate (logarithm transformed) of healthcare utilization. In **Model 1**, we denoted the two COVID-19 time segment(s) respectively as *seg_1* (week 103-116: March 17, 2020-June 15, 2020) and *seg_2* (week 140-152: November 27, 2020-Feburary 22, 2021); Y_{wk} was the counts of health services utilization in each week, assuming a Poisson distribution (or negative binomial distribution to handle potential over-dispersion) with person-year as offset; *wk* was a continuous variable indicating the calendar week (range:1-157) in the study period (April 1, 2018 - March 31, 2021); *seg_i* (*i*=1, 2) is a binary indicator (0 or 1) of the presence of the COVID-19 state of emergency; *wk_in_seg_i* was a continuous variable indicating the calendar week within *seg_i* (e.g., *wk_in_seg_1* = 103-116); and X_k indicates a group of covariates adjusted in the model. Since the outcome variable (Y_{wk}) was updated in each week for each patient, the generalized linear model (GLM) with mixed-effect was used to account for within-subject correlation of longitudinal, repeated measures of Y_{wk} over time. In this model, beta-coefficients (β_2, change of log rate, +/- indicates increase/decrease in rates) as well as percentage of changes (as exponential of β_2 -1, reported in the main text) in *seg_i* and their 95% CIs were computed after adjusting for covariates (X_k).

Table S2. The impact of COVID-19 emergency on health services utilization: results from stratified analyses

	β coef.*	95% CI	b coef.*	95% CI	*P*-value**
**Stratified analysis #1**	**Urban**		**Rural**		
**Hospitalization**					
	**Emerg. 1: March 17, 2020-June 15, 2020**				
change in log rate	-1.5	-2.24, -0.76	-2.49	-3.86, -1.13	0.23
	**Emerg. relaxed: June 16, 2020-November 26, 2020**				
change in log rate	-0.48	-1.21, 0.24	0.58	-0.90, 2.07	0.74
	**Emerg. 2: November 27, 2020-February 22, 2021**				
change in log rate	-0.77	-1.92, 0.38	-1.12	-3.16, 0.92	0.49
**ED visits**					
	**Emerg. 1: March 17, 2020-June 15, 2020**				
change in log rate	-2.46	-4.12, -0.81	-3.02	-5.26, -0.79	0.47
	**Emerg. relaxed: June 16, 2020-November 26, 2020**				
change in log rate	0.35	-1.20, 1.90	0.83	-1.61, 3.27	0.49
	**Emerg. 2: November 27, 2020-February 22, 2021**				
change in log rate	-0.95	-2.58, 0.67	-3.69	-6.93, -0.44	0.9
**Primary care visits**					
	**Emerg. 1: March 17, 2020-June 15, 2020**				
change in log rate	-1.03	-1.52, -0.54	-1.22	-2.08, -0.36	0.47
	**Emerg. relaxed: June 16, 2020-November 26, 2020**				
change in log rate	-0.47	-0.99, -0.04	-0.85	-1.71, 0.01	0.58
	**Emerg. 2: November 27, 2020-February 22, 2021**				
change in log rate	-0.88	-1.70, -0.05	-0.72	-2.05, 0.60	0.63
**Virtual care visits**					
	**Emerg. 1: March 17, 2020-June 15, 2020**				
change in log rate	1.31	0.78, 1.84	0.88	-0.44, 2.21	0.03
	**Emerg. relaxed: June 16, 2020-November 26, 2020**				
change in log rate	0.08	-1.80, 1.97	-0.54	-1.98, 0.91	0.8
	**Emerg. 2: November 27, 2020-February 22, 2021**				
change in log rate	-0.02	-0.37, 0.33	-0.23	-2.20, 1.74	0.58
**Stratified analysis #2**	**Diabetes with complications**		**Diabetes without complications**		
**Hospitalization**					
	**Emerg. 1: March 17, 2020-June 15, 2020**				
change in log rate	-1.48	-2.15, -0.82	-0.1	-2.24, 2.04	0.04
	**Emerg. relaxed: June 16, 2020-November 26, 2020**				
change in log rate	-0.67	-1.32, 0.02	-0.77	-2.97, 1.44	0.64
	**Emerg. 2: November 27, 2020-February 22, 2021**				
change in log rate	0.08	-1.35, 1.50	-1.16	-2.69, 0.36	0.73
**ED visits**					
	**Emerg. 1: March 17, 2020-June 15, 2020**				
change in log rate	-2.85	-4.39, -1.31	-2.17	-3.80, -0.54	0.08
	**Emerg. relaxed: June 16, 2020-November 26, 2020**				
change in log rate	0.64	-0.88, 2.16	0.71	-1.09, 2.51	0.49

		**Emerg. 2: November 27, 2020-February 22, 2021**			
change in log rate	-1.37	-2.97, 0.24	-1.45	-.3.43, 0.52	0.4
**Primary care visits**					
		**Emerg. 1: March 17, 2020-June 15, 2020**			
change in log rate	-1.36	-1.83, -0.88	-1.02	-1.05, -0.93	0.92
		**Emerg. relaxed: June 16, 2020-November 26, 2020**			
change in log rate	-0.39	-0.89, 0.11	-1.18	-2.12, -0.25	0.63
		**Emerg. 2: November 27, 2020-February 22, 2021**			
change in log rate	-1.08	-1.89, -0.27	0.1	-1.34, 1.54	0.23
**Virtual care visits**					
		**Emerg. 1: March 17, 2020-June 15, 2020**			
change in log rate	1.9	-1.91, 5.71	1.42	-0.38, 3.24	0.97
		**Emerg. relaxed: June 16, 2020-November 26, 2020**			
change in log rate	-1.01	-4.80, 2.77	-0.14	-1.81, 1.53	0.58
		**Emerg. 2: November 27, 2020-February 22, 2021**			
change in log rate	-0.82	-4.44, 2.78	1.25	-1.32, 3.83	0.33

* beta coefficient (change in log rate, +/- indicates increase/decrease in rates) was estimated using multivariable generalized linear regression models (GLM), after adjusting for seasonality (cos, sin), age, sex, ethnicity, education attainment, BMI categories, ever smoking (yes/no), physically active (yes/no), tertiles of the 2005 Canadian Healthy Eating Index, and the number of Elixhauser comorbidity at diagnosis.

** *p*-values indicates the differences between comparision groups (e.g., urban vs. rural).
